# Bis(μ-2,2′-bi-1*H*-imidazole-1,1′-di­acet­ato)bis­[diaqua­cobalt(II)] hexa­hydrate

**DOI:** 10.1107/S1600536809013701

**Published:** 2009-04-22

**Authors:** Tingting Zhang, Tao Zhang, Feng Xu, Hongze Liang

**Affiliations:** a State Key Laboratory Base of Novel Functional Materials and Preparation Science, Faculty of Materials Science and Chemical Engineering, Ningbo University, Ningbo 315211, People’s Republic of China

## Abstract

The dinuclear title compound, [Co_2_(C_10_H_8_N_4_O_4_)_2_(H_2_O)_4_]·6H_2_O, lies about an inversion centre. Each Co^II^ atom is six-coordinated by two water mol­ecules, two carboxyl­ate O atoms and two N atoms of two symmetry-related 2,2′-bi-1*H*-imidazole-1,1′-diacetate (*L* ^2−^) ligands in a slightly distorted octa­hedral geometry. Mol­ecules are linked into a three-dimensional framework *via* O—H⋯O and C—H⋯O hydrogen bonds.

## Related literature

For background to 2,2′-biimidazole derivatives, see: Atencio *et al.* (2004 ▶); Ghosh *et al.* (2006 ▶); Tadokoro & Nakasuji (2000 ▶); Zhang & Liang (2009 ▶). For the preparation of the ligand, see: Zhang *et al.* (2009 ▶).
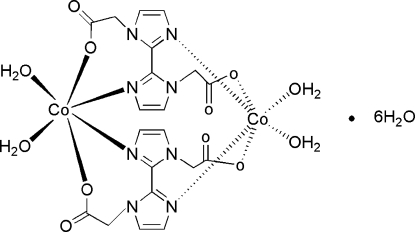


## Experimental

### 

#### Crystal data

[Co_2_(C_10_H_8_N_4_O_4_)_2_(H_2_O)_4_]·6H_2_O
*M* *_r_* = 794.43
Monoclinic, 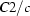

*a* = 15.902 (3) Å
*b* = 14.202 (3) Å
*c* = 14.998 (3) Å
β = 110.06 (3)°
*V* = 3181.7 (13) Å^3^
*Z* = 4
Mo *K*α radiation
μ = 1.13 mm^−1^
*T* = 295 K
0.50 × 0.42 × 0.18 mm

#### Data collection

Rigaku R-AXIS RAPID diffractometer
Absorption correction: multi-scan (*ABSCOR*; Higashi, 1995 ▶) *T*_min_ = 0.570, *T*_max_ = 0.813
15337 measured reflections
3625 independent reflections
3151 reflections with *I* > 2σ(*I*)
*R*_int_ = 0.029

#### Refinement

*R*[*F* ^2^ > 2σ(*F* ^2^)] = 0.027
*wR*(*F* ^2^) = 0.074
*S* = 1.10
3625 reflections
218 parameters
H-atom parameters constrained
Δρ_max_ = 0.41 e Å^−3^
Δρ_min_ = −0.29 e Å^−3^

### 

Data collection: *RAPID-AUTO* (Rigaku, 1998 ▶); cell refinement: *RAPID-AUTO*; data reduction: *CrystalStructure* (Rigaku/MSC, 2004 ▶); program(s) used to solve structure: *SHELXS97* (Sheldrick, 2008 ▶); program(s) used to refine structure: *SHELXL97* (Sheldrick, 2008 ▶); molecular graphics: *SHELXTL* (Sheldrick, 2008 ▶); software used to prepare material for publication: *SHELXL97*.

## Supplementary Material

Crystal structure: contains datablocks global, I. DOI: 10.1107/S1600536809013701/sj2607sup1.cif

Structure factors: contains datablocks I. DOI: 10.1107/S1600536809013701/sj2607Isup2.hkl

Additional supplementary materials:  crystallographic information; 3D view; checkCIF report

## Figures and Tables

**Table 1:** Selected geometric parameters (Å, °)

Co1—O3	2.0796 (13)
Co1—N4	2.1103 (14)
Co1—O6^i^	2.1108 (13)
Co1—N1^i^	2.1153 (15)
Co1—O4	2.1177 (14)
Co1—O5	2.1727 (14)

Symmetry code: (i) 
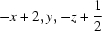
.

**Table 2:** Hydrogen-bond geometry (Å, °)

*D*—H⋯*A*	*D*—H	H⋯*A*	*D*⋯*A*	*D*—H⋯*A*
O4—H4*D*⋯O2^ii^	0.81	1.92	2.7309 (19)	171
O4—H4*C*⋯O8^iii^	0.93	1.93	2.855 (2)	173
O5—H5*D*⋯O9^iii^	0.83	1.94	2.752 (2)	169
O5—H5*C*⋯O7^iv^	0.99	1.90	2.888 (2)	170
O8—H8*C*⋯O6^v^	0.92	2.10	2.991 (2)	162
O8—H8*D*⋯O10^vi^	0.91	1.85	2.756 (3)	173
O9—H9*D*⋯O8	0.97	2.02	2.931 (3)	157
O9—H9*C*⋯O7^vi^	0.90	2.01	2.850 (2)	155
O10—H10*C*⋯O2^vii^	0.96	1.97	2.927 (3)	174
O10—H10*C*⋯O3^vii^	0.96	2.52	3.072 (2)	116
O10—H10*D*⋯O5^vii^	1.00	2.18	3.155 (3)	165
O10—H10*D*⋯O3^vii^	1.00	2.46	3.072 (2)	119

Symmetry codes: (ii) 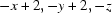; (iii) 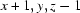; (iv) 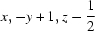; (v) 
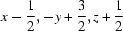
; (vi) 
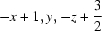
; (vii) 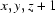.

**Fig. 1.**
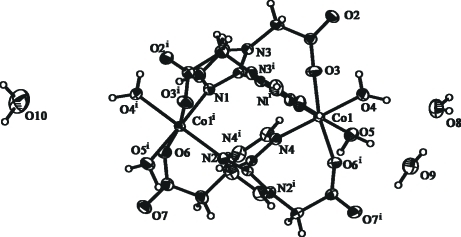
A view of (I), showing the labelling of the non-H atoms and 45% probability ellipsoids. H atoms have been omitted for clarity.

**Fig. 2.**
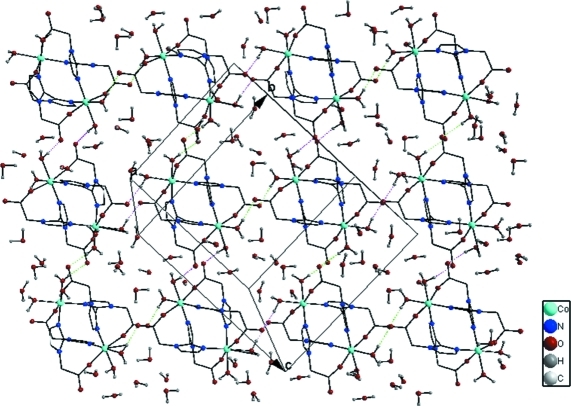
A perspective view of a two-dimensional sheet running parallel to [001], showing the packing mode and the O—H···O hydrogen-bonding interactions drawn as dashed lines. All H atoms not involved in the hydrogen-bond motifs have been omitted for clarity.

## supplementary crystallographic information

### Comment

Although N-substituted derivatives of 2,2'-biimidazole have recently been developed (Atencio *et al.*, 2004; Ghosh *et al.*, 2006; Tadokoro & Nakasuji, 2000; Zhang & Liang, 2009), few corresponding metal complexes have been reported. Here we report the crystal structure of Co_2_*L*_2_(H_2_O)_4_.6H_2_O [H_2_L = 2,2'-(2,2'-biimidazole-1,1'-diyl)diacetic acid]. The complex molecule lies about an inversion centre, as shown in Fig. 1, and the Co^II^ atoms show a slightly distorted octahedral geometry. The dihedral angle between the two imidazole rings of each *L*^2-^ is 72.50 (6)°. Each Co^II^ atom is six-coordinated by two O atoms from different monodentate carboxylate groups, two O atoms from the coordinating water molecules and two N atoms from two symmetry-related *L*^2-^ ligands. Selected bond distances and angles are listed in Table 1. The Co—O/N distances are in the ranges 2.0796 (13)–2.1727 (14) Å and 2.1103 (14)–2.1153 (15) Å, respectively. The Co···Co distance is 5.4881 Å, indicating that there is no interaction between the two metal centres.

An extensive network of O—H···O hydrogen bonds links the complex and water molecules to produce a number of substructures in two dimensions. A typical two-dimensional sheet, approximately parallel to [001], is shown in Fig. 2. Additional weak C—H···O hydrogen bonds (Table 2) generate a three-dimensional framework.

### Experimental

2,2'-(2,2'-Biimidazole-1,1'-diyl)diacetic acid (Zhang *et al.*, 2009) (0.1 g, 0.4 mmol) and Co(OH)_2_ (0.0372 g, 0.4 mmol) freshly prepared from CoCl_2_.6H_2_O and NaOH were added to distilled water (10 ml). The reaction mixture was adjusted to pH 6 with aqueous NaOH solution and stirred at room temperature for 20 min during which time a clear pink solution resulted. Red single crystals of (I) appeared within several weeks by slow evaporation at room temperature.

### Refinement

H atoms bound to C atoms were placed in geometrically calculated positions and were refined using a riding model, with *U*_iso_(H) = 1.2*U*_eq_(C). H atoms attached to O atoms were found in a difference Fourier synthesis and were refined using a riding model, with the O—H distances fixed as initially found and with *U*_iso_(H) values set at 1.5*U*eq(O).

### Crystal data

**Table:**

[Co_2_(C_10_H_8_N_4_O_4_)_2_(H_2_O)_4_]·6H_2_O	*F*(000) = 1640
*M**_r_* = 794.43	*D*_x_ = 1.658 Mg m^−^^3^
Monoclinic, *C*2/*c*	Mo *K*α radiation, λ = 0.71073 Å
Hall symbol: -C 2yc	Cell parameters from 15337 reflections
*a* = 15.902 (3) Å	θ = 3.1–27.4°
*b* = 14.202 (3) Å	µ = 1.13 mm^−^^1^
*c* = 14.998 (3) Å	*T* = 295 K
β = 110.06 (3)°	Block, red
*V* = 3181.7 (13) Å^3^	0.50 × 0.42 × 0.18 mm
*Z* = 4	

### Data collection

**Table:**

Rigaku R-AXIS RAPID diffractometer	3625 independent reflections
Radiation source: fine-focus sealed tube	3151 reflections with *I* > 2σ(*I*)
graphite	*R*_int_ = 0.029
ω scans	θ_max_ = 27.4°, θ_min_ = 3.1°
Absorption correction: multi-scan (*ABSCOR*; Higashi, 1995)	*h* = −20→19
*T*_min_ = 0.570, *T*_max_ = 0.813	*k* = −18→18
15337 measured reflections	*l* = −19→19

### Refinement

**Table:**

Refinement on *F*^2^	Secondary atom site location: difference Fourier map
Least-squares matrix: full	Hydrogen site location: inferred from neighbouring sites
*R*[*F*^2^ > 2σ(*F*^2^)] = 0.027	H-atom parameters constrained
*wR*(*F*^2^) = 0.074	*w* = 1/[σ^2^(*F*_o_^2^) + (0.0275*P*)^2^ + 4.2044*P*] where *P* = (*F*_o_^2^ + 2*F*_c_^2^)/3
*S* = 1.10	(Δ/σ)_max_ = 0.002
3625 reflections	Δρ_max_ = 0.41 e Å^−^^3^
218 parameters	Δρ_min_ = −0.29 e Å^−^^3^
0 restraints	Extinction correction: *SHELXL97* (Sheldrick, 2008), Fc^*^=kFc[1+0.001xFc^2^λ^3^/sin(2θ)]^-1/4^
Primary atom site location: structure-invariant direct methods	Extinction coefficient: 0.0061 (3)

### Special details

**Table:**

Geometry. All e.s.d.'s (except the e.s.d. in the dihedral angle between two l.s. planes) are estimated using the full covariance matrix. The cell e.s.d.'s are taken into account individually in the estimation of e.s.d.'s in distances, angles and torsion angles; correlations between e.s.d.'s in cell parameters are only used when they are defined by crystal symmetry. An approximate (isotropic) treatment of cell e.s.d.'s is used for estimating e.s.d.'s involving l.s. planes.
Refinement. Refinement of *F*^2^ against ALL reflections. The weighted *R*-factor *wR* and goodness of fit *S* are based on *F*^2^, conventional *R*-factors *R* are based on *F*, with *F* set to zero for negative *F*^2^. The threshold expression of *F*^2^ > σ(*F*^2^) is used only for calculating *R*-factors(gt) *etc*. and is not relevant to the choice of reflections for refinement. *R*-factors base. 0 d on *F*^2^ are statistically about twice as large as those based on *F*, and *R*- factors based on ALL data will be even larger.

### Fractional atomic coordinates and isotropic or equivalent isotropic displacement parameters (Å^2^)

**Table:**

	*x*	*y*	*z*	*U*_iso_*/*U*_eq_	
Co1	1.023692 (15)	0.749865 (14)	0.077182 (15)	0.01916 (9)	
N1	0.91453 (10)	0.82568 (10)	0.29571 (10)	0.0224 (3)	
N2	0.88351 (10)	0.58280 (10)	0.21753 (10)	0.0243 (3)	
N3	0.88985 (10)	0.91865 (10)	0.17141 (10)	0.0248 (3)	
N4	0.93715 (9)	0.67422 (10)	0.13078 (9)	0.0209 (3)	
O2	0.89286 (9)	0.98537 (9)	−0.05853 (9)	0.0285 (3)	
O3	0.92283 (9)	0.84716 (9)	0.01511 (9)	0.0323 (3)	
O4	1.11054 (11)	0.82618 (9)	0.02372 (11)	0.0389 (4)	
H4D	1.1043	0.8825	0.0295	0.047*	
H4C	1.1409	0.8131	−0.0181	0.047*	
O5	0.96590 (9)	0.67292 (9)	−0.05502 (9)	0.0319 (3)	
H5D	1.0017	0.6536	−0.0797	0.038*	
H5C	0.9304	0.6160	−0.0524	0.038*	
O6	0.87182 (9)	0.64981 (8)	0.38539 (9)	0.0280 (3)	
O7	0.87743 (10)	0.50471 (9)	0.44485 (9)	0.0344 (3)	
O8	0.19082 (11)	0.79054 (12)	0.88353 (11)	0.0472 (4)	
H8C	0.2507	0.7990	0.8941	0.057*	
H8D	0.1575	0.8073	0.8230	0.057*	
O9	0.10278 (12)	0.61000 (12)	0.88740 (11)	0.0501 (4)	
H9D	0.1444	0.6586	0.8843	0.060*	
H9C	0.1196	0.5660	0.9336	0.060*	
O10	0.89665 (17)	0.84232 (17)	0.80245 (13)	0.0832 (7)	
H10C	0.8954	0.8928	0.8449	0.100*	
H10D	0.9155	0.7956	0.8558	0.100*	
C1	0.95208 (11)	0.87378 (11)	0.24321 (11)	0.0198 (3)	
C2	0.82375 (12)	0.83991 (14)	0.25327 (13)	0.0301 (4)	
H2A	0.7800	0.8144	0.2743	0.036*	
C6	0.95635 (11)	0.62664 (11)	0.21149 (11)	0.0187 (3)	
C7	0.81313 (12)	0.60473 (14)	0.13716 (14)	0.0324 (4)	
H7A	0.7541	0.5850	0.1218	0.039*	
C3	0.80770 (13)	0.89635 (14)	0.17667 (13)	0.0317 (4)	
H3A	0.7521	0.9161	0.1357	0.038*	
C8	0.84668 (12)	0.66100 (13)	0.08439 (13)	0.0282 (4)	
H8A	0.8137	0.6868	0.0258	0.034*	
C4	0.90598 (15)	0.98220 (12)	0.10244 (13)	0.0312 (4)	
H4B	0.9627	1.0140	0.1324	0.037*	
H4A	0.8595	1.0298	0.0849	0.037*	
C5	0.90808 (11)	0.93378 (12)	0.01265 (12)	0.0221 (3)	
C9	0.88299 (15)	0.51645 (13)	0.29141 (14)	0.0329 (4)	
H9A	0.9370	0.4786	0.3080	0.039*	
H9B	0.8324	0.4743	0.2657	0.039*	
C10	0.87762 (11)	0.56101 (12)	0.38158 (12)	0.0240 (3)	

### Atomic displacement parameters (Å^2^)

**Table:**

	*U*^11^	*U*^22^	*U*^33^	*U*^12^	*U*^13^	*U*^23^
Co1	0.02548 (14)	0.01487 (13)	0.01840 (13)	0.00047 (8)	0.00914 (9)	0.00001 (8)
N1	0.0240 (7)	0.0230 (7)	0.0228 (7)	0.0021 (6)	0.0112 (6)	0.0027 (6)
N2	0.0274 (7)	0.0254 (7)	0.0227 (7)	−0.0072 (6)	0.0119 (6)	−0.0030 (6)
N3	0.0320 (8)	0.0233 (7)	0.0214 (7)	0.0078 (6)	0.0119 (6)	0.0055 (6)
N4	0.0218 (7)	0.0216 (7)	0.0185 (6)	−0.0003 (5)	0.0058 (5)	0.0003 (5)
O2	0.0384 (7)	0.0241 (6)	0.0231 (6)	−0.0005 (5)	0.0105 (5)	0.0048 (5)
O3	0.0421 (8)	0.0207 (6)	0.0303 (7)	0.0092 (5)	0.0076 (6)	0.0011 (5)
O4	0.0662 (10)	0.0198 (6)	0.0475 (8)	−0.0039 (6)	0.0412 (8)	−0.0004 (6)
O5	0.0413 (8)	0.0264 (7)	0.0284 (7)	−0.0011 (6)	0.0125 (6)	−0.0068 (5)
O6	0.0312 (7)	0.0204 (6)	0.0337 (7)	−0.0046 (5)	0.0129 (6)	−0.0023 (5)
O7	0.0513 (9)	0.0243 (6)	0.0302 (7)	−0.0067 (6)	0.0173 (6)	0.0013 (5)
O8	0.0448 (9)	0.0605 (10)	0.0420 (8)	0.0034 (8)	0.0223 (7)	0.0040 (8)
O9	0.0700 (11)	0.0437 (9)	0.0418 (9)	0.0050 (8)	0.0260 (8)	0.0003 (7)
O10	0.1034 (17)	0.1029 (17)	0.0366 (10)	0.0215 (14)	0.0152 (11)	−0.0105 (10)
C1	0.0246 (9)	0.0172 (7)	0.0190 (7)	0.0025 (6)	0.0091 (6)	0.0002 (6)
C2	0.0239 (9)	0.0388 (10)	0.0316 (9)	0.0046 (8)	0.0145 (7)	0.0050 (8)
C6	0.0215 (8)	0.0153 (7)	0.0199 (7)	−0.0008 (6)	0.0080 (7)	−0.0015 (6)
C7	0.0213 (8)	0.0408 (11)	0.0335 (10)	−0.0072 (8)	0.0075 (7)	−0.0082 (8)
C3	0.0264 (9)	0.0390 (10)	0.0295 (9)	0.0116 (8)	0.0091 (8)	0.0044 (8)
C8	0.0229 (8)	0.0342 (10)	0.0237 (8)	0.0009 (7)	0.0032 (7)	−0.0017 (7)
C4	0.0508 (12)	0.0193 (8)	0.0260 (9)	0.0080 (8)	0.0163 (8)	0.0070 (7)
C5	0.0207 (8)	0.0219 (8)	0.0228 (8)	0.0022 (6)	0.0062 (6)	0.0021 (7)
C9	0.0479 (12)	0.0218 (8)	0.0364 (10)	−0.0103 (8)	0.0241 (9)	−0.0018 (8)
C10	0.0236 (8)	0.0228 (8)	0.0269 (8)	−0.0067 (7)	0.0103 (7)	−0.0019 (7)

### Geometric parameters (Å, °)

**Table:**

Co1—O3	2.0796 (13)	O6—C10	1.267 (2)
Co1—N4	2.1103 (14)	O6—Co1^i^	2.1108 (13)
Co1—O6^i^	2.1108 (13)	O7—C10	1.242 (2)
Co1—N1^i^	2.1153 (15)	O8—H8C	0.9180
Co1—O4	2.1177 (14)	O8—H8D	0.9119
Co1—O5	2.1727 (14)	O9—H9D	0.9684
N1—C1	1.329 (2)	O9—H9C	0.9028
N1—C2	1.378 (2)	O10—H10C	0.9636
N1—Co1^i^	2.1153 (15)	O10—H10D	1.0029
N2—C6	1.345 (2)	C1—C1^i^	1.467 (3)
N2—C7	1.370 (2)	C2—C3	1.351 (3)
N2—C9	1.457 (2)	C2—H2A	0.9300
N3—C1	1.347 (2)	C6—C6^i^	1.470 (3)
N3—C3	1.373 (2)	C7—C8	1.356 (3)
N3—C4	1.460 (2)	C7—H7A	0.9300
N4—C6	1.327 (2)	C3—H3A	0.9300
N4—C8	1.379 (2)	C8—H8A	0.9300
O2—C5	1.248 (2)	C4—C5	1.523 (2)
O3—C5	1.251 (2)	C4—H4B	0.9700
O4—H4D	0.8143	C4—H4A	0.9700
O4—H4C	0.9320	C9—C10	1.522 (3)
O5—H5D	0.8252	C9—H9A	0.9700
O5—H5C	0.9945	C9—H9B	0.9700
			
O3—Co1—N4	90.29 (6)	H10C—O10—H10D	91.9
O3—Co1—O6^i^	169.21 (5)	N1—C1—N3	111.15 (15)
N4—Co1—O6^i^	96.47 (5)	N1—C1—C1^i^	125.05 (16)
O3—Co1—N1^i^	96.53 (6)	N3—C1—C1^i^	123.68 (16)
N4—Co1—N1^i^	94.46 (6)	C3—C2—N1	110.01 (16)
O6^i^—Co1—N1^i^	91.32 (6)	C3—C2—H2A	125.0
O3—Co1—O4	89.63 (6)	N1—C2—H2A	125.0
N4—Co1—O4	179.80 (5)	N4—C6—N2	111.28 (14)
O6^i^—Co1—O4	83.63 (6)	N4—C6—C6^i^	125.21 (15)
N1^i^—Co1—O4	85.36 (6)	N2—C6—C6^i^	123.35 (16)
O3—Co1—O5	84.59 (6)	C8—C7—N2	106.34 (16)
N4—Co1—O5	87.97 (5)	C8—C7—H7A	126.8
O6^i^—Co1—O5	87.26 (6)	N2—C7—H7A	126.8
N1^i^—Co1—O5	177.31 (5)	C2—C3—N3	106.24 (16)
O4—Co1—O5	92.21 (6)	C2—C3—H3A	126.9
C1—N1—C2	105.27 (14)	N3—C3—H3A	126.9
C1—N1—Co1^i^	129.17 (12)	C7—C8—N4	109.68 (16)
C2—N1—Co1^i^	125.45 (12)	C7—C8—H8A	125.2
C6—N2—C7	107.33 (15)	N4—C8—H8A	125.2
C6—N2—C9	125.47 (16)	N3—C4—C5	114.20 (15)
C7—N2—C9	126.89 (16)	N3—C4—H4B	108.7
C1—N3—C3	107.32 (14)	C5—C4—H4B	108.7
C1—N3—C4	126.82 (16)	N3—C4—H4A	108.7
C3—N3—C4	125.82 (16)	C5—C4—H4A	108.7
C6—N4—C8	105.35 (14)	H4B—C4—H4A	107.6
C6—N4—Co1	129.08 (11)	O2—C5—O3	125.60 (16)
C8—N4—Co1	125.51 (11)	O2—C5—C4	115.77 (15)
C5—O3—Co1	140.80 (12)	O3—C5—C4	118.62 (15)
Co1—O4—H4D	110.1	N2—C9—C10	115.06 (15)
Co1—O4—H4C	135.1	N2—C9—H9A	108.5
H4D—O4—H4C	112.2	C10—C9—H9A	108.5
Co1—O5—H5D	115.8	N2—C9—H9B	108.5
Co1—O5—H5C	116.2	C10—C9—H9B	108.5
H5D—O5—H5C	103.6	H9A—C9—H9B	107.5
C10—O6—Co1^i^	128.25 (12)	O7—C10—O6	125.95 (17)
H8C—O8—H8D	110.6	O7—C10—C9	115.27 (15)
H9D—O9—H9C	120.2	O6—C10—C9	118.76 (15)

Symmetry codes: (i) −*x*+2, *y*, −*z*+1/2.

### Hydrogen-bond geometry (Å, °)

**Table:**

*D*—H···*A*	*D*—H	H···*A*	*D*···*A*	*D*—H···*A*
O4—H4D···O2^ii^	0.81	1.92	2.7309 (19)	171
O4—H4C···O8^iii^	0.93	1.93	2.855 (2)	173
O5—H5D···O9^iii^	0.83	1.94	2.752 (2)	169
O5—H5C···O7^iv^	0.99	1.90	2.888 (2)	170
O8—H8C···O6^v^	0.92	2.10	2.991 (2)	162
O8—H8D···O10^vi^	0.91	1.85	2.756 (3)	173
O9—H9D···O8	0.97	2.02	2.931 (3)	157
O9—H9C···O7^vi^	0.90	2.01	2.850 (2)	155
O10—H10C···O2^vii^	0.96	1.97	2.927 (3)	174
O10—H10C···O3^vii^	0.96	2.52	3.072 (2)	116
O10—H10D···O5^vii^	1.00	2.18	3.155 (3)	165
O10—H10D···O3^vii^	1.00	2.46	3.072 (2)	119

Symmetry codes: (ii) −*x*+2, −*y*+2, −*z*; (iii) *x*+1, *y*, *z*−1; (iv) *x*, −*y*+1, *z*−1/2; (v) *x*−1/2, −*y*+3/2, *z*+1/2; (vi) −*x*+1, *y*, −*z*+3/2;  (vii) *x*, *y*, *z*+1.
